# Draft Genome Sequence of Delftia tsuruhatensis Strain 45.2.2, Colonizer of Zebrafish, Danio rerio, Skin Mucus

**DOI:** 10.1128/mra.00666-22

**Published:** 2022-10-18

**Authors:** Allie Chaires, Kayla Thomas, Anne M. Estes, Anna K. S. Jozwick

**Affiliations:** a Center for Natural Sciences, Goucher College, Baltimore, Maryland, USA; b Department of Biological Sciences, Towson University, Baltimore, Maryland, USA; University of Southern California

## Abstract

Delftia tsuruhatensis strain 45.2.2 is a member of the zebrafish, Danio rerio, skin mucus microbiota. Its genome is similar in size and GC content to those of other *Delftia* strains.

## ANNOUNCEMENT

Delftia tsuruhatensis strain 45.2.2 was sequenced to determine its potential role as a member of the healthy adult zebrafish, Danio rerio, skin mucus community. Initially isolated from sludge (1), Delftia tsuruhatensis strains have been found in water systems, in fish guts, and as an opportunistic human pathogen (1–3). It is a Gram-negative rod. We are interested in whether this strain encodes antimicrobial and quorum quenching capabilities, as described previously for other D. tsuruhatensis strains (4, 5).

*D. tsuruhatensis* strain 45.2.2 was collected in June 2019 by swabbing the skin mucus of healthy laboratory-reared zebrafish as described previously (6) and approved by the Goucher College IACUC. Dilutions were plated onto Reasoner's 2A agar medium and incubated for 48 h at 28°C. Picked colonies were purify streaked 3 times for isolation on tryptic soy agar (TSA) at 28°C to 30°C and immediately stored as a glycerol stock. Bacterial colony DNA was isolated using a modified boil prep method followed by PCR amplification of the 16S rRNA gene and Sanger sequencing of the amplicon (https://doi.org/10.6084/m9.figshare.21203093). The negative-control sample produced no amplicon. Genewiz (Plainfield, NJ) performed Sanger sequencing on the purified products. The 16S rRNA gene sequences were trimmed manually using SnapGene Viewer (version 5.0.4; GSL Biotech LLC, San Diego, CA) and compared to the NCBI BLAST database. Identical 16S rRNA gene sequences from 5 isolates cultured from 3 different fish were identified as Delftia tsuruhatensis, with the sequence under NCBI accession number NR_113870.1 being the best match.

*D. tsuruhatensis* strain 45.2.2 was chosen as a representative isolate (NCBI accession number ON603637). The strain was grown from a glycerol stock on TSA at 28°C, and the plate was processed by the Microbial Genome Sequencing Center (MiGS) in Pittsburgh, PA (https://www.migscenter.com). MiGS isolated genomic DNA using the Zymo DNA miniprep kit with bead-beating lysis (Zymo Research, Tustin, CA). A library was made using the Illumina DNA prep kit and sequenced on an Illumina NextSeq 2000 platform. A total of 6,687,850 paired 151-bp reads were generated from 992,098,997 bp of raw sequence data. During demultiplexing, MiGS trimmed off the Illumina adapter sequences. We analyzed the sequences using the KBase.us narrative interface set to default parameters unless otherwise specified. Trimmomatic v0.36 (7) set to a tail crop length of 150 bp, a head crop length of 15 bp, a leading and trailing minimum quality of 3, and a minimum read length of 130 bp produced good-quality reads as assessed using FastQC v0.11.9 (8). Assemblies were made in both Velvet v1.2.10 (9) and SPAdes v3.15.3 (10), with Quast v4.4 (11, 12) identifying the SPAdes assembly as being of a higher quality, with 91 contigs having an *N*_50_ value of 123,371, a GC content of 66.59%, and 150× coverage. The SPAdes assembly was annotated using the NCBI Prokaryotic Genome Annotation Pipeline (PGAP) (13–15). CheckM v1.0.18 (16) determined that the 6,613,850-nucleotide genome was 100.0% complete, with only 0.72% contamination. This high-quality draft genome had 5,973 coding genes and 6,080 total genes, 2 CRISPR arrays, 1 complete rRNA gene (5S, 16S, and 23S), and 47 tRNAs. GTDB-Tk v1.7.0 (17) classified the isolate as Comamonas tsuruhatensis. This genus has since been renamed *Delftia* (18).

### Data availability.

The sequence reads (SRA accession number SRX15699338), the assembly (GenBank genome accession number JAMXHV000000000), and BioSample accession number SAMN29042291 are associated with BioProject accession number PRJNA849171.
